# Endosperm transfer cell-specific genes and proteins: structure, function and applications in biotechnology

**DOI:** 10.3389/fpls.2014.00064

**Published:** 2014-02-27

**Authors:** Sergiy Lopato, Nikolai Borisjuk, Peter Langridge, Maria Hrmova

**Affiliations:** Australian Centre for Plant Functional Genomics, University of AdelaideGlen Osmond, SA, Australia

**Keywords:** endosperm transfer cells, biotechnology, invertase, lipid transfer protein, two-component system

## Abstract

Endosperm transfer cells (ETC) are one of four main types of cells in endosperm. A characteristic feature of ETC is the presence of cell wall in-growths that create an enlarged plasma membrane surface area. This specialized cell structure is important for the specific function of ETC, which is to transfer nutrients from maternal vascular tissue to endosperm. ETC-specific genes are of particular interest to plant biotechnologists, who use genetic engineering to improve grain quality and yield characteristics of important field crops. The success of molecular biology-based approaches to manipulating ETC function is dependent on a thorough understanding of the functions of ETC-specific genes and ETC-specific promoters. The aim of this review is to summarize the existing data on structure and function of ETC-specific genes and their products. Potential applications of ETC-specific genes, and in particular their promoters for biotechnology will be discussed.

## INTRODUCTION

**FIGURE 1:**
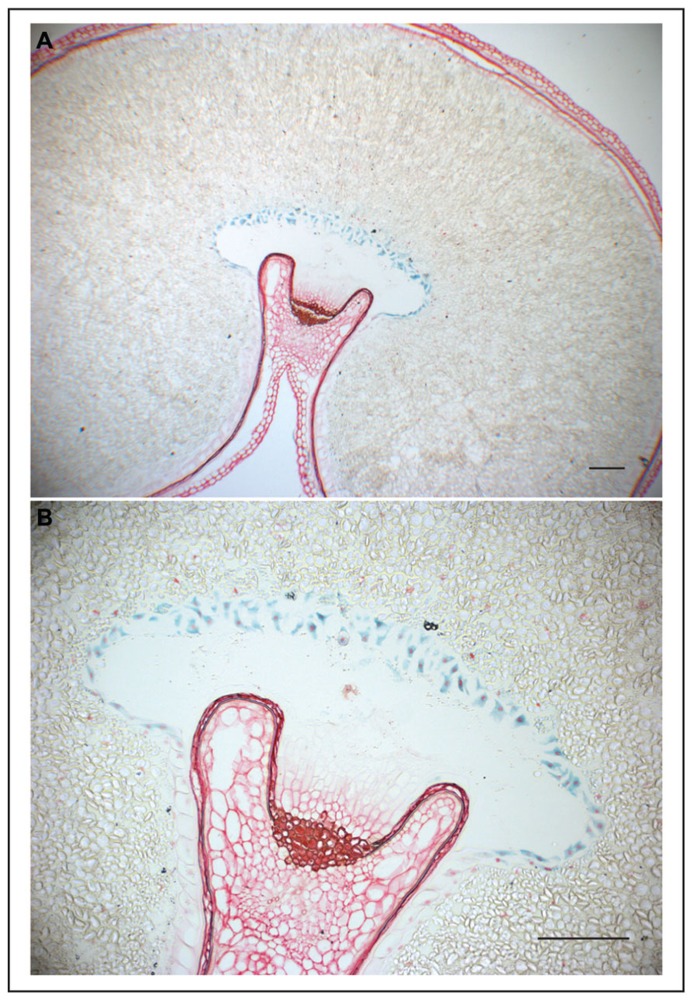
**GUS expression in wheat grain directed by the ETC-specific *TdPR60* promoter.** **(A)** Histochemical GUS assay counterstained with safranin in 10 μm thick transverse sections of transgenic wheat cariopsis at 31 DAP. **(B)** A detailed view on ETC at larger magnification. Bars = 200 μm.

Transfer cells are highly specialized plant cells responsible for the transport of solutes and nutrients from source to sink organs (Offler et al., 2003; Olsen, 2004). They can be found at many plant exchange surfaces, including phloem loading zones within the root, and unloading zones for transfer of nutrients to the endosperm in developing seeds. Endosperm transfer cells (ETC) have easily recognizable structural features, including an elongated shape and numerous cell wall in-growths, which greatly increase the surface area of the cell membrane and consequently enhance transport of solutes (Olsen, 2004). In maize seeds, transfer cells are located at the base of the endosperm. By contrast, in wheat and barley they are positioned along the crease (**Figure 1**; Olsen, 2004; Monjardino et al., 2013). Various molecular markers based on genes that are specifically or preferentially expressed in ETC, have been identified and isolated from maize, wheat, barley, and rice (Hueros et al., 1995, 1999a; Doan et al., 1996; Serna et al., 2001; Cai et al., 2002; Gutierrez-Marcos et al., 2004; Li et al., 2008; Kovalchuk et al., 2009). Initially, some of these markers were used by cytologists to localize ETC and determine their fate at different stages of grain development. For instance, it was discovered that ETC are not a part of maternal tissues, but rather a modification of part of the aleurone cell layer(s), which is located near to maternal vascular tissues. The identity of ETC is defined irreversibly during syncytium development and cellularization, the earliest stages of endosperm development (Costa et al., 2003; Olsen, 2004).

More recently, the function of some ETC-specific marker genes was elucidated, and their involvement in ETC differentiation and function established (Carlson et al., 2000; Weschke et al., 2003; Wang et al., 2008b; Muñiz et al., 2010). All currently known ETC-specific genes and those predominantly expressed in ETC cells, can be classified into one of the five groups (**Table 1**): (1) signal receptors and transducers, forming the basis of a two-component signaling system for ETC differentiation and development; (2) transcriptional regulators and co-factors; (3) genes responsible for sugar conversion and transport; (4) genes encoding lipid transfer proteins (LTPs); and (5) genes encoding proteins with as yet unknown functions. Since the grain filling process is dependent on ETC structure and function, there is a high level of interest from biotechnologists in genes involved in the formation and function of ETC. This review will summarize current knowledge of the function of ETC-specific genes and the molecular structure of their products, focussing on commercially important grass species (i.e., maize, wheat, and barley), but also including relevant molecular evidence from the model plant *Arabidopsis*. Potential applications for some ETC-specific genes in genetic engineering for improved grain size, quality, and yield under favorable conditions and also under environmental stresses, will be discussed.

**Table 1:** Genes that are specifically or predominantly expressed in endosperm transfer cells^*^.

Plant	Gene name	Protein type	GenBank Accession	Suggested function^**^	Cited
*Zea mays*	*ZmTCRR-1*	Type-A response regulator	AM085299	Signal transduction	Muñiz et al. (2006)
*Zea mays*	*ZmTCRR-2*	Type-A response regulator	Unavailable^***^	Signal transduction	Muñiz et al. (2010)
*Zea mays*	*ZmMRP-1*	One-repeat MYB TF	AJ318518	Transcriptional activation of ETC/BETL-specific genes	Gómez et al. (2002)
*Zea mays*	*ZmMRPI-1*	C2H2 zinc finger protein	NM_001153367	Negative regulator of ZmMRP-1	Royo et al. (2009)
*Zea mays*	*ZmMRPI-2*	C2H2 zinc finger protein	NM_001158398	Negative regulator of ZmMRP-1	Royo et al. (2009)
*Zea mays*	*INCW2*	Cell-wall invertase	AF165180	Converting sucrose into glucose and fructose	Carlson et al. (2000)
*Sorgum bicolor*	*SbINCW*	Cell-wall invertase	EF177465 (partial CDS)	Converting sucrose into glucose and fructose	Jain et al. (2008b)
*Oriza sativa*	*GIF1*	Cell-wall invertase	EU095553	Converting sucrose into glucose and fructose	Wang et al. (2008a)
*Hordeum vulgare*	*HvCWINV1*	Cell-wall invertase	AJ534447	Converting sucrose into glucose and fructose	Weschke et al. (2003)
*Gossypium hirsutum*	*GhCWIN1*	Cell-wall invertase	AI725433^****^	Converting sucrose into glucose and fructose	Wang and Ruan (2012)
*Hordeum vulgare*	*HvSF6FT1*	Sucrose:fructan 6-fructosyltransferase (soluble invertase)	X83233	Converting sucrose into glucose and fructose	Weschke et al. (2003)
*Hordeum vulgare*	*HvSTP1*	Hexose transporter	AJ534445	Importing hexoses into the endosperm	Weschke et al. (2003)
*Zea mays*	*MEG1*	LTP, defensin type	AY536121	Protection against pathogens	Gutierrez-Marcos et al. (2004)
*Zea mays*	*BETL-1*	LTP, defensin type	JQ420076	Protection against pathogens	Hueros et al. (1995)	
*Zea mays*	*BETL-2 *(*BAP2*)	LTP	AJ133529	Signal transduction, protection against pathogens	Hueros et al. (1999a)	
*Zea mays*	*BETL-3*	LTP, defensin type	AJ133530	Protection against pathogens	Hueros et al. (1999a)
*Zea mays*	*BETL-4*	LTP, Bowman-Birk family of α-amylase/trypsin inhibitors	AJ133531	Protection against insects and pathogens	Hueros et al. (1999a)
*Oriza sativa*	*OsPR9a*	LTP, defensin type	EU264060	Protection against insects and pathogens	Li et al. (2008)
*Hordeum vulgare*	*END1*	nsLTP	Z69631	Signal transduction, protection against pathogens	Doan et al. (1996)
*Oriza sativa*	*OsPR602*	nsLTP	EU264061	Signal transduction, protection against pathogens	Li et al. (2008)
*Triticum aestivum*	*TaPR60*	nsLTP	EU264062	Signal transduction, protection against pathogens	Kovalchuk et al. (2009)
*Oriza sativa*	*AL1*	Anthranilate N-hydroxycinnamoyl/ benzoyltransferase	Os01g0382200 (Rice Annotation Project Database)	Function in plants is unknown	Kuwano et al. (2011)

* Genes in the **Table 1** are arranged according to the group specification in the text.

** Current knowledge of the function of ETC-specific genes is dependent on available data, and thus in some instances function of ETC-specific genes is speculative.

*** Sequence originates from a private cDNA collection.

**** The EST sequence used for cloning a full-length CDS. The full length CDS sequence was not provided by Wang and Ruan (2012).

## TWO COMPONENT SIGNALING PLAYS AN IMPORTANT ROLE IN DIFFERENTIATION OF ETC

**FIGURE 2:**
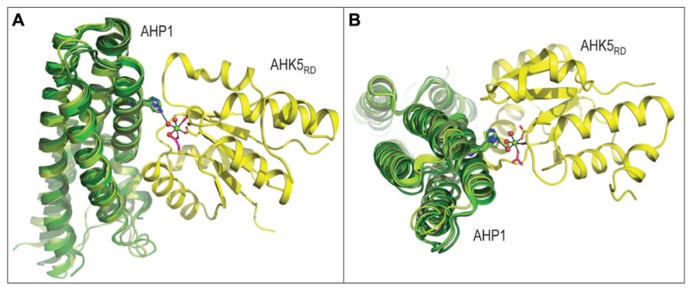
**Three-dimensional structure of the AHK5_**RD**_-AHP1 complex from *Arabidopsis thaliana* (PDB 4EUK), consisting of the histidine-containing phosphotransfer (AHP1, green) and kinase (AHK5_**RD**_, yellow) region, is shown in two orthogonal orientations.** The structure of the complex in panel **A** is rotated by approximately 90 degrees to produce a view shown in panel **B**. The mechanism of intermolecular phosphotransfer mediated by the *Arabidopsis* AHK5_RD_-AHP1 complex. Maize ZmHP2 (PDB 1WN0, smudge green), *Medicago truncatula* MtHPT1 (PDB 3US6, limon green) and rice OsHPT (PDB 1YVI, forest green) are superposed over the *Arabidopsis* AHP1. The His in AHP1 and Asp in AHK5_RD_ residues that respectively donate and accept a phosphoryl group are shown in sticks in atomic green and yellow colors, respectively. The octahedral coordination geometry of Mg^2^^+^ (green sphere) participating in the phosphotransfer reaction is indicated by black dashes (atomic distances between 1.9 Å and 2.0 Å), where Mg^2^^+^ is coordinated by Asp from AHK5_RD_, three water molecules (red spheres) and two other residues (Asp and Cys) of AHK5_RD_. The distance of 3.4 Å between His from AHP1 and one of the water molecules is also shown.

Two component signaling (TCS) was initially discovered in 1981 for bacteria (Hall and Silhavy, 1981), and its involvement in nearly all signal transduction events has been demonstrated. Existence of TCS in plants was revealed for the first time in 1996 (Kakimoto, 1996). The first type of TCS components described in plants are membrane-localized receptor histidine kinases (HK), responsible for the perception of signals transferred by ligand molecules, usually hormones. The binding of a ligand molecule leads to auto-phosphorylation of the receptor domain and intra-molecular transfer of the phosphoryl residue to the receiver domain of the HK (Hwang and Sheen, 2001). This is followed by phosphate transfer to a small soluble histidine phospho-transfer protein (HP), which is able to move to the nucleus. The structural characteristics of the AHK5_RD_-AHP1 complex from *Arabidopsis thaliana* (Bauer et al., 2013), suggest the process for transfer of the phosphoryl group from AHK5_RD_ to AHP1 (**Figure 2**). HP proteins from maize (Sugawara et al., 2005), *Medicago truncatula* (Ruszkowski et al., 2013) and rice (Wesenberg et al., unpublished data, PDB 1YVI) superimposed over the AHP1 protein from *Arabidopsis* indicate that HP acceptor proteins from diverse plant species fold similarly, and that interfaces between HP and kinases are highly conserved (**Figure 2**). Further, comparison of the level of conservation of residues at the binding interface region of 22 HP proteins from 16 plant species including those from *Arabidopsis*, reveals a remarkably high level of preservation of architecture in HP proteins; in particular the spatial positions of a key His residue. It is therefore expected that the mode of action of the AHK5_RD_-AHP1 complex serves as a paradigm to understand the function of TCS in higher plants at the molecular level (Bauer et al., 2013). Analogous machineries of intermolecular phosphotransfers are likely to operate in both mono- and dicotyledonous plants. In the nucleus, HP activates type-B response regulators (RR), which are a subfamily of MYB transcription factors (TF). Members of this MYB subfamily in turn activate target genes, including genes encoding the type-A RR, which are usually negative regulators of hormone signaling pathways (Hwang and Sheen, 2001).

Two component signaling is involved in a range of plant developmental processes and responses to stresses and other stimuli, such as the development of meristems (Kim et al., 2006), maintenance of circadian rhythms (Mizuno, 2005), senescence (Riefler et al., 2006), phosphate and nitrogen availability responses (Sakakibara et al., 1998; Coello and Polacco, 1999; Takei et al., 2001, 2002), sulfur metabolism processes (Fernandes et al., 2009), responses to heavy metals (Srivastava et al., 2009), and other abiotic (Chefdor et al., 2006; Jain et al., 2008a; Karan et al., 2009) and biotic (Jolivet et al., 2007) stresses. Recently, many TCS components were identified in ETC, confirming ETC as the primary mediator of signal transduction between maternal tissue and developing grain (Muñiz et al., 2006, 2010; Thiel et al., 2012).

**FIGURE 3:**
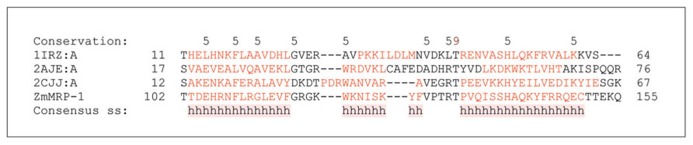
**Domain analyses of selected DNA binding proteins containing MYB domains that are involved in the two component system (TCS).** A multiple sequence alignment of ZmMRP-1 involved in TCS with three MYB domain-containing proteins ARR10-B of the GARP family from *Arabidopsis thaliana* (PDB 1IRZ, chain A), a telomeric repeat-binding protein from *Arabidopsis thaliana* (PDB 2AJE, chain A), and a MYB domain of the RAD transcription factor from *Antirrhinum majus *(PDB 2CJJ, chain A). Protein sequences were aligned with ProMals3D (Pei et al., 2008) and analysed for domain boundaries using ProDom (Bru et al., 2005). The predicted and consensus secondary structures (ss) are shown in red (α-helices, h) and black (loops) types. Conservation of residues on a scale of 9–5 is shown at the top of the diagram. The absolutely conserved and similar residues are highlighted in brown and black, respectively.

The first TCS components identified in cereal grains were the maize genes *Transfer Cell Response Regulators* *1* and* 2 *(*ZmTCRR-1* and *ZmTCRR-2*; **Table 1**). These encode members of the type-A RR of the TCS, which are responsible for phospho-transfer-based signal transduction (Muñiz et al., 2006, 2010). The *TCRR* genes were found to be expressed exclusively in the ETC layer 8–14 days after pollination (DAP), when transfer-cell differentiation is most active. However, the ZmTCRR-1 protein was also detected in conductive tissue deep inside the endosperm, where transcription of the gene was not observed (Muñiz et al., 2006). This finding suggests that TCS is involved in intercellular signal transduction. A possible role of TCRR proteins is to integrate external signals with seed developmental processes (Muñiz et al., 2006, 2010). The promoter of ZmTCRR-1 was strongly *trans*-activated in heterologous systems by the transfer cell-specific TF ZmMRP-1, which is a MYB type TF (Muñiz et al., 2006, 2010; Gomez et al., 2009; **Figure 3**).

Recently, the ETC layer was isolated by laser micro-dissection and pressure catapulting (LMPC) from barley grains at different stages of development (Thiel et al., 2012). Sequence analysis of the barley ETC transcriptome revealed a large number of TCS components. Practically all known components of the TCS were identified and in some cases several types of each component were evident. For example, among the HK identified were six putative ethylene receptors, two putative cytokinin receptors and three HK of unknown function with high similarity to kinases from rice and *Arabidopsis* (Thiel et al., 2012). Six genes encoding HPs were also found to be expressed in the ETC layers. Two of these contained no His residue in the HP domain. All types (A, B, and C) of RRs were found in barley ETC. Three type-A RRs had higher levels of sequence similarity to rice RRs than to either ETC-specific ZmTCRR-1 or ZmTCRR-2 from maize (Muñiz et al., 2010), which cluster separately in phylogenetic analyzes. 11 sequences of type-B RRs and four isoforms of type-C RRs were also present in the barley ETC transcriptome (Thiel et al., 2012). The high number and high mRNA abundance of TCS components in developing ETC suggests that TCS is crucial for ETC development and consequently for grain filling.

## TRANSCRIPTIONAL REGULATION OF ETC FORMATION AND FUNCTION

ZmMRP-1 is so far the only transfer cell-specific TF to have been identified and characterized in cereals (**Table 1**). It is proposed to play a central role in the regulatory pathways controlling transfer cell differentiation and function (Gomez et al., 2009). *ZmMRP-1* is a single-copy gene that encodes proteins with a MYB-related DNA binding domain (**Figure 3**) and a nuclear localization signal. Analysis of domain boundaries in the ZmMRP-1 protein reveals the location of a MYB-like domain sequence (**Figure 3**). The MYB-like domain in ZmMRP-1 shows significant similarity to secondary structural element distributions in known MYB domain-containing proteins, including ARR10-B of the GARP family from *A. thaliana* (Hosoda et al., 2002), a telomeric repeat-binding protein from *A. thaliana* (Sue et al., 2006) and a MYB domain of the RAD TF from *Antirrhinum majus *(Stevenson et al., 2006; **Figure 3**). In developing maize grain, * ZmMRP-1* transcript was detected in the cytoplasmic region of the basal endosperm coenocytes as early as 3 DAP. Because the transfer cell layer develops from this part of maize coenocytes, it is reasonable to propose a role for *ZmMRP-1* in ETC formation. However, the strongest *ZmMRP-*1 expression was observed in transfer cell layers from 3 to 16 DAP and peaked at 11 DAP, when formation of ETC was already completed, suggesting an additional role of this TF in ETC function (Gomez et al., 2009).

To evaluate the level of conservation of ETC formation and function in different plant species, spatial expression patterns of *ZmMRP-1* were studied in transgenic lines of maize, *Arabidopsis*, tobacco and barley which were transformed with a *ZmMRP-1* promoter-GUS reporter construct (Barrero et al., 2009). GUS signal was detected in several plant organs in regions of active transport between source and sink tissues and at vascular connection sites between developing organs and the main plant vasculature. Promoter induction was observed in all tested species at early developmental stages of transport-to-sink tissues, including in the ETC layer (Barrero et al., 2009). Based on these results it was proposed that ETC differentiate in a similar way in diverse plant species, and that this differentiation is initiated by conserved induction signals. Using both *in planta* and yeast experiments it was demonstrated that *ZmMRP-1* promoter activity is modulated by different carbohydrates. Glucose was found to be the most effective inducer of the *ZmMRP-1* promoter (Barrero et al., 2009).

Several target genes of *ZmMRP-1* have been identified (Gómez et al., 2002; Costa et al., 2004; Gutierrez-Marcos et al., 2004; Barrero et al., 2006; Muñiz et al., 2006, 2010). The activation of BETL (*Basal Endosperm Transfer Layer*) gene promoters was initially demonstrated by co-transformation of two constructs in tobacco protoplasts. In these experiments, constitutive expression of *ZmMRP-1* led to the activation of a co-transformed GUS gene driven by the promoter from the *Basal Endosperm Transfer Layer* (*BETL-1*) gene (Barrero et al., 2006). In whole plants, it was also shown that ectopic expression of *ZmMRP-1* under the control of the ubiquitin promoter in BETL-1:GUS transgenic maize led to activation of ETC-specific gene expression (Gómez et al., 2002). In a separate study, the promoter of *MATERNALLY EXPRESSED GENE1* (*MEG1*) was found to be activated by ZmMRP-1, when the transcriptional *MEG1* promoter-GUS fusion construct and a transcriptional 35S:MRP1 construct were co-transformed into tobacco protoplasts (Costa et al., 2004; Gutierrez-Marcos et al., 2004). Since *MEG1* is expressed in maize basal transfer cells from 10 to 20 DAP, it can potentially be activated by ZmMRP-1 in maize plants. The promoter of the ETC-specific gene encoding a type-A RR, ZmTCRR-1, was also strongly activated in heterologous systems by ZmMRP-1 (Muñiz et al., 2006, 2010).

Interaction between ZmMRP-1 and the promoter of the transfer cell specific gene *BETL-1*, led to activation of the *BETL-1* promoter in various cell types (Barrero et al., 2006). Although the reporter construct containing the *BETL-1* promoter was silent in all tested types of cells when transformed alone, transient co-expression of ZmMRP-1 led to significant activation of the reporter gene. This suggests that ZmMRP-1 does not require the help of ETC-specific factors for promoter activation. The transient expression system was used to find specific *cis*-elements in the *BETL-1* promoter. A *cis*-element consisting of a 12 bp motif containing two consecutive repeats (2 × TATCTC) was situated approximately 100 bp upstream of the TATA box of the *BETL-1* promoter. Specific binding of ZmMRP-1 to this *cis*-element was confirmed *in vitro* using electrophoretic mobility shift experiments (Barrero et al., 2006). Similar *cis*-elements were found in several other transfer cell-specific promoters and were designated as “transfer cell box” elements. However, the “transfer cell box” was not identified in ETC-specific promoters from wheat or rice (Li et al., 2008; Kovalchuk et al., 2009). In these species, a single copy of the TATCTC motif was found in a number of ETC-specific promoters, suggesting that there has been degeneration of transfer cell box sequences during evolution of some grass species. It cannot be excluded that ETC-specific expression of some genes is regulated by other, as yet unidentified TF(s), or at least requires the presence of other factors for specific interaction with promoter sequences.

**FIGURE 4:**
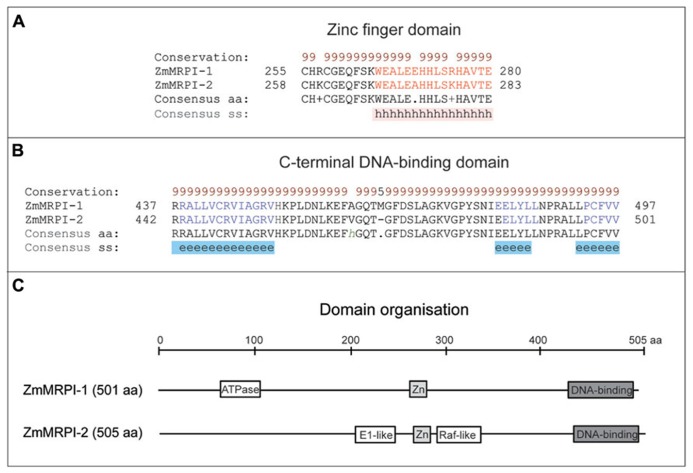
**Domain analyses of ZmMRPI-1 and ZmMRPI-2 proteins involved in the two component system (TCS) contain a highly conserved Zinc finger domain in nearly the same location.** **(A)** A sequence alignment of the Zn finger domains, which fold into α-helices. **(B)** A sequence alignment of the C-terminal DNA-binding domains, which fold into sheets. Protein sequences were aligned with ProMals3D (Pei et al., 2008) and analysed for domain boundaries using SMART (Letunic et al., 2012) and ProDom (Bru et al., 2005). The predicted and consensus secondary structures (ss) are shown in panels **(A)** and **(B)** in red (α-helices, h), blue (β-sheets, e) and black (loops) types. Conservation of residues (brown and black types) on a scale of 9–5 is shown at the top of the diagram. **(C)** Schematics of domain organization of ZmMRPI-1 and ZmMRPI-2, as analysed by SMART (Letunic et al., 2012) and ProDom (Bru et al., 2005). A position of the ATPase, central region-like domain is shown in ZmMRPI-1, while in ZmMRPI-2, glycoprotein E1-like and Raf-like Ras-binding domains are schematically represented. In both entries Zn finger- (light gray) and C-terminal DNA-binding (dark gray) domains are also illustrated. The schematic is drawn to scale of 505 amino acid (aa) residues.

It has been shown that ZmMRP-1 TF binds not only to gene promoters, but it may also bind other proteins (Royo et al., 2009). Two proteins were isolated in a yeast 2-hybrid screen using full length ZmMRP-1 as bait; these were designated as ZmMRP-1 Interactors 1 and 2 (ZmMRPI-1 and ZmMRPI-2; **Table 1**). Binding of ZmMRP-1 to ZmMRPI-1 and ZmMRPI-2 was confirmed *in planta *by co-localization of the proteins in transfer cell nuclei. ZmMRPI-1 and ZmMRPI-2 are very similar proteins, both belonging to the C(2)H(2) zinc finger protein subfamily of nuclear proteins. Members of this subfamily interact with MYB-related TF through their C-terminal conserved domains (Royo et al., 2009). In ZmMRPI-1 and ZmMRPI-2 proteins, a Zinc finger domain of the C2H2-type and a C-terminal DNA-binding domain are highly conserved both in disposition and in sequence identities at the amino acid level, which are 89 and 97% for the Zinc finger and DNA-binding domains, respectively (**Figure 4**). In both proteins the Zinc finger (**Figure 4A**) and C-terminal DNA-binding (**Figure 4B**) domains fold into α-helices, and β-sheets, respectively. Although the full-length sequences of ZmMRPI-1 and ZmMRPI-2 share very high sequence identity (85%) and similarity (94%), analysis using the SMART database (Letunic et al., 2012) identified an ATPase, central region-like domain in ZmMRPI-1, but a glycoprotein E1-like domain and putative Raf-like Ras-binding domain in ZmMRPI-2 (**Figure 4C**). These domains were positioned in different locations of the protein sequences, reflecting localized differences in amino acid sequence that may be important for specific regulatory functions.

It was shown that MRPI proteins can modulate activation of ETC-specific promoters by interacting with ZmMRP-1 (Royo et al., 2009). In addition, the expression of *MRPI* genes in maize and *Arabidopsis* are found to be expressed at the same nutrient exchange surfaces, where the expression of *ZmMRP-1* has been previously detected. *MRPI*-orthologs genes have been identified in the rice and *Arabidopsis *genomes (Royo et al., 2009).

Although there are no reports of other types of TF which are specifically or predominantly expressed in ETC and participate in the regulation of ETC development and function, the existence of such TFs cannot be excluded. Most members of the HD-Zip IV TF subfamily, for example, are expressed in grain tissues (Yang et al., 2002; Javelle et al., 2011; Kovalchuk et al., 2012a), and are involved in regulation of LTP expression (Javelle et al., 2010; Lopato et al., unpublished data). It has been reported that at least three wheat LTPs (TaLtp7.2a, TaLtp9.1a, and TaLtp9.3e) are specifically expressed in the main vascular bundle of wheat scutellum (Boutrot et al., 2007), which plays a major role in sugar transport from endosperm to embryo during seed germination. Recently, it was demonstrated that the wheat HD-Zip IV TF TdGL9H1 is also predominantly expressed in the scutellar vascular bundle and thus is potentially a regulator of these LTPs (Kovalchuk et al., 2012b). Since several LTPs have been found to be specifically expressed in ETC layers (Hueros et al., 1995, 1999a; Doan et al., 1996; Li et al., 2008; Kovalchuk et al., 2009), it would be reasonable to predict that at least one member of HD-Zip IV subfamily is also specifically or predominantly expressed in ETC and involved in the transcriptional regulation of at least some ETC-specific LTPs.

## GENES RESPONSIBLE FOR SUGAR TRANSPORT TO ENDOSPERM

Sucrose is the main carbohydrate transported from photosynthetically active tissues to sinks such as root, flower, and seed (Ruan et al., 2010; Lemoine et al., 2013). However, sucrose does not enter in this form, but is converted into the hexoses glucose and fructose. These reactions are catalyzed by sugar invertases (INVs), which are reported also to have regulatory roles in plant growth and development (LeClere et al., 2008, 2010; Chourey et al., 2010). INVs can be classified into three groups according to their localisation in cells: vacuolar (VIN), cytoplasmic (CIN) and cell wall-bound or apoplastic (CWIN or INCW). Several studies have demonstrated that INCWs are the major type of INVs responsible for the delivery of hexoses to the developing seed (**Table 1**). Tight regulation of the delivery of hexoses by CWINs provides a mechanism for controlling cell division and even cell differentiation in developing kernels (Miller and Chourey, 1992; Weber et al., 1996). A positive correlation between seed development and the activity of INCWs has been observed in faba bean (Weber et al., 1995, 1996), maize (Cheng et al., 1996; Vilhar et al., 2002; Chourey et al., 2006), barley (Weschke et al., 2003; Sreenivasulu et al., 2004), rice (Hirose et al., 2002; Wang et al., 2008a), tomato (Zanor et al., 2009), and cotton (Wang and Ruan, 2012). In addition to INVs localized at the interface of sink organs, hexose transporters facilitate the import of hexoses into seed endosperm and other sinks (Bihmidine et al., 2013; reviewed in Slewinski, 2011).

### ETC-SPECIFIC CELL WALL-BOUND INVERTASES

Transfer cells are the gateway for sugar transport from maternal tissue to the endosperm. Sugar delivery in turn directly affects transfer cell formation. Mutants of the maize gene *Miniature1* (*mn1*) show an anatomical lesion in the pedicel region and reduced size of the kernel (Lowe and Nelson, 1946; Miller and Chourey, 1992; Cheng et al., 1996). Kernel size is reduced due to reductions in both mitotic activity and cell size (Vilhar et al., 2002). *Miniature1* encodes a cell wall invertase (INCW2) that was originally thought to be localized in the basal endosperm and pedicel (Miller and Chourey, 1992; Cheng et al., 1996; Carlson et al., 2000). However, histochemical visualization of invertase activity in the maternal pedicel region revealed that *INCW2* is expressed exclusively in the ETC layer, and that the pedicel is served by the orthologs gene *INCW1* (Chourey et al., 2006).

Loss of *INCW2* function in the *mn1 *mutant led to reduced size and number of the labyrinth-like wall-in-growths (WIGs) of ETC, a subsequent decrease in plasma membrane surface area and decline in ETC transport capacity, and consequently reduced grain filling (Kang et al., 2009). Analysis of intracellular structure by electron microscopy revealed that in the *mn1* mutant, WIGs in the ETC were stunted and the endoplasmic reticulum in these cells was swollen; Golgi density in the mutant ETC was reduced to 51% of the Golgi density in wild-type plants (Kang et al., 2009). INCW2-specific immunogold particles were detected in WIGs, the endoplasmic reticulum, Golgi stacks, and the trans-Golgi network in the ETC of wild-type plants, but were extremely rare in the ETC of the *mn1* mutant (Kang et al., 2009).

A recent study was undertaken to identify gene products that are metabolically regulated in ETC in response to invertase deficiency (Silva-Sanchez et al., 2013). Comparisons of soluble and cell wall-bound proteomes of the *mn1* mutant and wild-type (*Mn1*) plants revealed 131 differentially expressed proteins, which fell into two major groups: proteins related to carbohydrate metabolic and catabolic processes, or proteins involved in cell homeostasis (Silva-Sanchez et al., 2013).

Developing kernels of the* mn1* mutant also have drastically reduced auxin (IAA) levels (LeClere et al., 2008). The reduced IAA levels are due to decreased transcript abundance of the *ZmYucca1* (*ZmYuc1*) gene. This gene encodes flavin monooxygenase, a key enzyme in the IAA biosynthetic pathway (Chourey et al., 2010; LeClere et al., 2010). Using two different approaches it was shown that expression of *ZmYuc1* is regulated by sugar levels (LeClere et al., 2010). These data explain how sugar levels can influence auxin levels in seed, which in turn regulates specific aspects of seed development.

A similar role of INCW genes in seed development in rice has been reported (Wang et al., 2008b). Rice seed weight was increased by overexpression of the *GRAIN INCOMPLETE FILLING 1* (*GIF1*) gene that encodes a cell-wall invertase (Wang et al., 2008b). Interestingly, although expression under the native *GIF1* promoter increased grain size, ectopic expression of the *GIF1* gene under the 35S or rice *Waxy* promoters resulted in smaller grains. This observation illustrates that transgenic plant phenotypes depend on the spatial and temporal patterns of transgene expression.

A study of barley cell wall-bound invertase genes revealed an expression pattern similar to expression of maize *INCW2*. Two cell wall-bound invertase genes, *HvCWINV1 *and *HvCWINV2*, were preferentially expressed in the maternal-basal endosperm boundary just before cellularization (Weschke et al., 2003). Transcripts of *HvCWINV1* were localized within the first row of endosperm cells, in the outermost area of the nucellar projection as well as in ETC before starch filling. *HvCWINV2* is expressed early in development, predominantly in the style region and later on in pericarp areas which transiently accumulate starch (Weschke et al., 2003).

Possible additional roles for INCWs have been revealed by examining spatial and temporal expression of the *GhCWIN1* gene in cotton seeds during very early seed development, from just before fertilization to the beginning of starch accumulation in the endosperm (Wang and Ruan, 2012). The dynamics of *GhCWIN1* expression suggest an involvement of INSWs in regulating endosperm nuclear division, embryonic provascular formation and differentiation of ETC (Wang and Ruan, 2012).

### ETC-SPECIFIC CELL SOLUBLE INVERTASES

A barley gene encoding the soluble acid invertase enzyme, sucrose:fructan 6-fructosyltransferase (HvSF6FT1), shows similar temporal and spatial expression patterns to *HvCWINV2* (Weschke et al., 2003). *HvSF6FT1 *is expressed in the inner cell layers of maternal pericarp above the dorsal cells, at 3 DAP. At 4 DAP the expression of *HvSF6FT1* is observed in the ventral pericarp and ETC. At 6 DAP, expression of this gene is limited to the ETC layers (Weschke et al., 2003). *HvSF6FT1* transcript levels and acid soluble invertase activity were found to be highest in the maternal pericarp 1–2 days after flowering (DAF). *HvSF6FT1* is strongly expressed in regions flanking the main vascular bundle and to a lesser extent in filial ETC, which continues until grain maturity (Weschke et al., 2003).

### AN ETC-SPECIFIC HEXOSE TRANSPORTER

**FIGURE 5:**
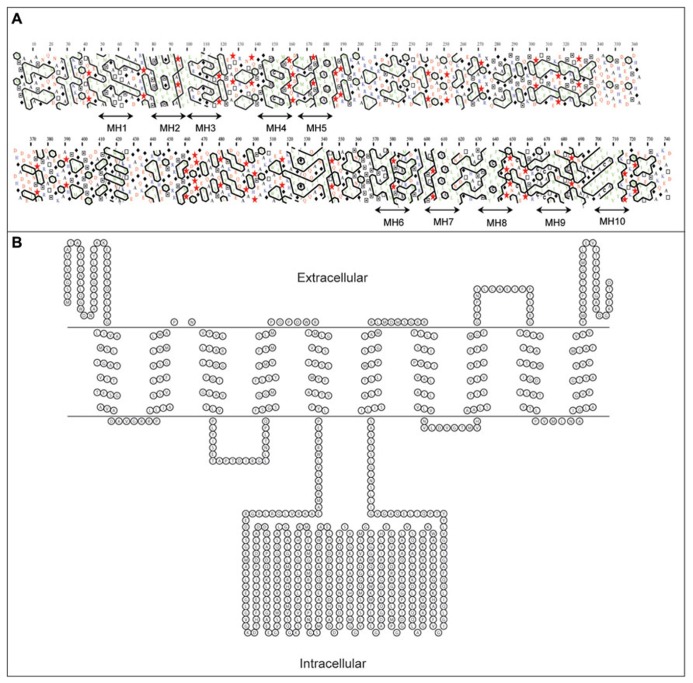
**Secondary structure analyses of a barley hexose transporter HvSTP1, **(A)** A bi-dimensional hydrophobic cluster analysis (HCA) plot (Callebaut et al., 1997).** Positions of 10 membrane helices MH1–MH10 are marked by arrowed lines. Proline residues are shown as red stars, glycine residues as black diamonds, serine residues are empty squares and threonine residues are shown as squares containing a black dot in the center. Negatively charged residues are colored in red and positively charged residues are in blue. Other residues are shown by their single amino acid letter codes. The amino acid numbers are read from the top to the bottom of the plots (in duplicate) in a left to right direction. **(B)** A topology model predicted by PRED-TMR algorithm (Pasquier et al., 1999). The intracellular and extracellular positions of individual domains are shown. The topology map was drawn with TOPO (http://www.sacs.ucsf.edu/TOPO/topo.html).

Regions of the developing barley grain associated with *HvCWINV1* expression are also associated with expression of a hexose transporter, HvSTP1 (Weschke et al., 2003; **Table 1**). HvSTP1 is expressed at a very low level within the pericarp, but much more highly in the syncytial endosperm at 3 DAF and in ETC at 7 DAF. The temporal and spatial association of expression of HvsSTP1 and INVs suggests that hexoses released by INVs within the endospermal cavity are transferred by the transporter to the liquid part of the mitotically active endosperm (Weschke et al., 2003). HvSTP1 is a large membrane protein of 743 amino acid residues with up to 10 trans-membrane α-helices, as assessed by hydrophobic cluster analysis (HCA; Callebaut et al., 1997) and PRED-TMR (Pasquier et al., 1999; **Figure 5A**). Our topological analysis shows that HvSTP1 harbors a large intracellular module rich in hydrophilic residues that are positioned approximately in the middle of an α-helical bundle (**Figure 5B**).

Thus, the activity of cell wall INVs establishes a sucrose concentration gradient between maternal symplast and endosperm apoplast by hydrolysis of sucrose to fructose and glucose moieties. Subsequently, an ETC-specific hexose transporter facilitates the import of hexoses into the endosperm (Miller and Chourey, 1992; Cheng et al., 1996; Weschke et al., 2003).

## ETC-SPECIFIC LIPID TRANSFER PROTEINS

### PLANT NON-SPECIFIC LIPID TRANSFER PROTEINS

Non-specific lipid transfer proteins (nsLTPs) have been found in a broad range of tissues from plants, animals and fungi (Crain and Zilversmit, 1980; Tai and Kaplan, 1985; Kader, 1996; Ng et al., 2012). The term “non-specific” indicates that LTPs can bind with phospholipids or their derivatives of broad specificity (Ostergaard et al., 1993). In plants, nsLTPs form multigenic families of structurally related proteins with low levels of protein sequence identity. All plant nsLTPs are originally translated as precursor proteins and contain hydrophobic signal peptides of different length, which are subsequently proteolytically processed by endopeptidases. The enzymes responsible for the processing of precursors remain largely unknown but are likely to be members of the subtilase group of Ser proteases (Murphy et al., 2012). The precise place and role of signal peptide processing of nsLTPs is also unknown. However, in one example it was shown that signal peptide processing takes place in microsomal membranes (Bernhard et al., 1991). It is not yet clear whether the proteolytic cleavage of a signal peptide also occurs in other types of membranes.

Plant nsLTPs usually have a molecular mass between 6.5 and 10.5 kDa and an isoelectric point ranging between 8.5 and 12 (Jose-Estanyol et al., 2004). Each mature nsLTP sequence usually contains a characteristic 8-cysteine residue motif: Cys_1_-Xn-Cys_2_-Xn-Cys_3_Cys_4_-Xn-Cys_5_XCys_6_-Xn-Cys_7_-Xn-Cys_8_. Plant nsLTPs were initially classified into two types, based on their size and localization (Kader, 1996). Later, a new classification based on the analysis of a large number of nsLTPs sequences was proposed, to include nine types (I–IX) of nsLTPs (Boutrot et al., 2008). Genome-wide analysis revealed 49 *Arabidopsis*, 52 rice and 156 wheat nsLTPs (Boutrot et al., 2008). Recently, the first plant nsLTP database (nsLTPDB^1^) was constructed, which initially contained 595 nsLTPs from 121 different species. This database includes information about LTP sequence, protein structure, relevant references and also some biological data (Wang et al., 2012).

Plant nsLTPs are involved in embryogenesis (Sterk et al., 1991), defense against bacterial and fungal pathogens (Molina and Garcia-Olmedo, 1993; Molina et al., 1993; Hendriks et al., 1994; Lindorff-Larsen and Winther, 2001), symbiosis (Krause et al., 1994; Pii et al., 2009), plant response to environmental stresses (White et al., 1994; Liu et al., 2000; Cameron et al., 2006) and in the delivery of waxes to cuticle (Hendriks et al., 1994; Lindorff-Larsen and Winther, 2001; Lee et al., 2009). It has also been postulated that nsLTPs can associate with hydrophobic cell wall compounds and disrupt or facilitate cell wall extension (Nieuwland et al., 2005). A role in these very diverse functions is based on the ability of nsLTPs to carry a broad range of hydrophobic molecules such as fatty acids or fatty acid derivatives (Garcia-Olmedo et al., 1995). LTPs can catalyze the exchange of lipids between natural and artificial membranes *in vitro* (Helmkamp, 1986; Wirtz and Gadella, 1990; Kader, 1996). A role of nsLTPs in intracellular lipid transfer has also been proposed (Miquel et al., 1988) but not yet proven. Existing knowledge suggests that LTPs are secreted from cells into the extracellular (cell wall) space (Serna et al., 2001; Yeats and Rose, 2008). Precise mechanisms of uploading, delivery to membranes and cuticle, and unloading of lipidic molecules by LTPs remain unclear.

### LTP GENES SPECIFICALLY OR PREDOMINANTLY EXPRESSED IN ETC

Most nsLTP genes show very specific spatial patterns of expression, and several ETC-specific genes encoding different nsLTPs have been identified in developing kernels (Hueros et al., 1995, 1999b; Doan et al., 1996; Cai et al., 2002; Gutierrez-Marcos et al., 2004; Li et al., 2008; Kovalchuk et al., 2009; **Table 1**). Four types of nsLTPs are found in maize BETL. BETL-1 and BETL-3 show sequence homology to defensin-like proteins; BETL-2 has no homologous sequences; and BETL-4 shares some homology with the Bowman-Birk family of α-amylase/trypsin inhibitors (Hueros et al., 1995, 1999b). Members of these protein families have been shown to inhibit the growth of fungi and bacteria (Broekaert et al., 1997). Defensins and probably other types of LTPs can alter the permeability of fungal plasma membranes and hence may act as regulators of transport through plant cellular membranes (Thompson et al., 2001). Proteolytic processing and secretion into adjacent maternal tissue of BETL-2 (also named BAP2) protein was demonstrated by immunolocalization in different grain tissues (Serna et al., 2001). A gene with sequence similarity to *BETL-3*, *OsPR9a*, has been found in rice. However, *OsPR9a* expression is not restricted to ETC, but was also detected in some rice floral tissues (Li et al., 2008).

Expression of BETL-1 and BETL-2 proteins was found to be strongly reduced in the maize *reduced grain filling1* (*rgf1*)**mutant, which also showed decreased uptake of sugars in endosperm cells at 5–10 DAP and changes in pedicel development (Maitz et al., 2000). The *rgf1* mutant is morphologically similar to the *mni1* mutant; it causes up to 70% reduction of grain filling in maize. Starch accumulation (but not synthesis) is reduced in *rgf1* kernels. Therefore, the *Rgf1* gene, which has not been identified, may be involved in sugar sensing or transport in ETC (Maitz et al., 2000).

Expression of at least some ETC-specific genes is under maternal control. One such gene, *Maternally Expressed Gene1* (*MEG1*), encodes a LTP which bears structural similarity to defensins (Gutierrez-Marcos et al., 2004). *MEG1* is expressed exclusively in the BETL cells of maize endosperm and has a parent-of-origin expression pattern during early stages of endosperm development. However, at later stages of endosperm development it shows biallelic expression. The product of this gene is glycosylated and localizes to the labyrinthine in-growths of the walls of transfer cells (Gutierrez-Marcos et al., 2004).

**FIGURE 6:**
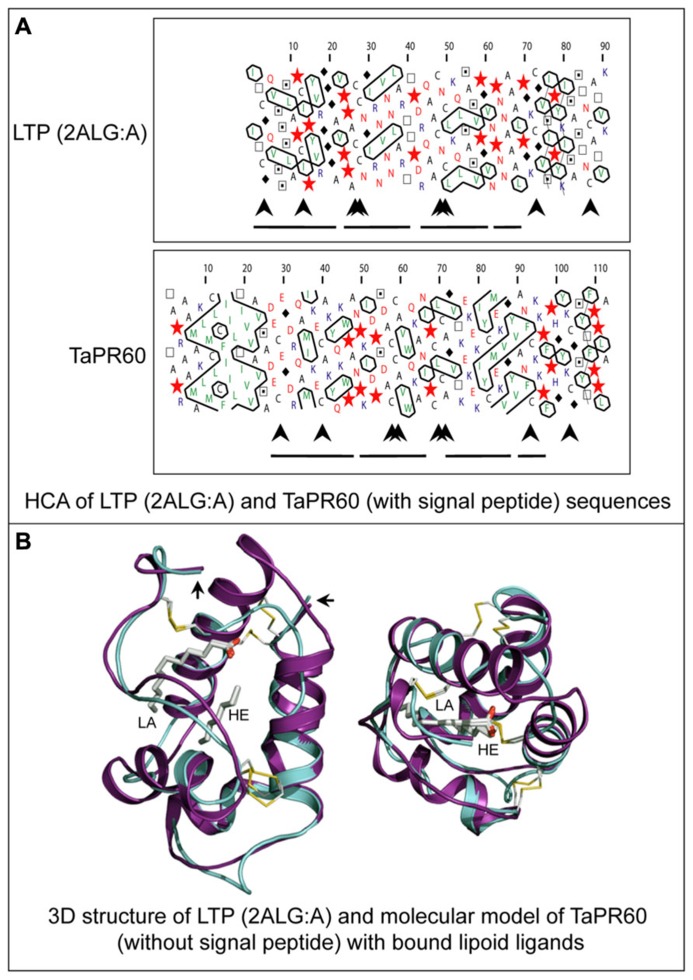
**Structural molecular modeling of the TaPR60 lipid transfer protein.** **(A)** HCA of a non-specific lipid transfer protein (LTP) from *Prunus persica *(PDB 2ALG:A) and of TaPR60. Positions of N-terminal hydrophobic signal peptide (*large* *arrow*), four paired conserved cysteines (*arrowheads*) and α-helices (*lines*) are marked. **(B)** Superposition of the TaPR60 model (*cyan*) on the template crystal structure of 2ALG:A (*magenta*) showing distribution of the secondary structural elements. A root-mean-square-deviation value for 69 structurally equivalent residues is 1.5 Å over the Cα backbone positions. The dispositions of bound lauric acid (LA; *left*) and heptane (HE; *right*), in *cpk* colors internalized in protein cavities, and the positions of four invariant disulfide bridges (*yellow*) are also shown. The *right-hand-side* and *left-hand*-*side *arrows indicate N- and C-terminal parts of both proteins, respectively. The Figure was modified from Kovalchuk et al. (2009).

Another class of ETC-specific nsLTP genes was identified in the barley transfer cell domain of the endosperm coenocyte. The gene was designated *Endosperm 1* (*END1*; Doan et al., 1996). The expression pattern of the barley *END1* gene and its ortholog from wheat were studied using *in situ* hybridization (Doan et al., 1996; Drea et al., 2005). Before cellularization *END1* transcripts accumulate mainly in the coenocyte above the nucellar projection, and after cellularization in the ventral endosperm over the nucellar projection. At 8 DAP and later, a low level of *END1 *expression can be detected in the ETC and the adjacent starchy endosperm (Doan et al., 1996). The function of *END1 *remains unknown. The expression of the wheat *END1* homologue, designated *TaPR60*, was studied using transgenic wheat, barley, and rice stably transformed with a gene promoter-GUS fusion construct (Kovalchuk et al., 2009). In wheat and barley, *TaPR60* is expressed predominantly in ETC and in the adjacent starchy endosperm. However, in rice the expression pattern of *TaPR60 *was rather different, suggesting that the regulatory mechanisms for ETC-specific expression in rice are different to wheat and barley (Kovalchuk et al., 2009). A molecular model of the TaPR60 protein lacking its N-terminal hydrophobic peptide, constructed using the crystal structure of a non-specific LTP from *Prunus persica *as a template (**Figure 6**), indicates the likely positions of a fatty acid (lauric acid) and lipid-mimicking molecule (heptane). These are enclosed in the central cavity of a triple α-helical bundle of TaPR60. Modeling supports the hypothesis that TaPR60 is involved in binding and transfer of lipid molecules. It was also shown that the cavity of TaPR60 retains its shape both with and without the hydrophobic signal peptide (Kovalchuk et al., 2009). Therefore, TaPR60 could potentially enclose the lipid and lipid-like molecule(s) in the cavity during precursor processing and secretion. One of the possible functions of TaPR60 could be in mediation of lipid delivery to or through a membrane (Kovalchuk et al., 2009). Similar findings were reported for a closely related protein, TdPR61, isolated from durum wheat (*T. durum*; Kovalchuk et al., 2012a).

Expression directed by the promoter of a rice homologue of the *END1 *gene, *OsPR602*, was studied in transgenic rice and barley. In rice, the promoter of *OsPR602* was active in ETC and above the ETC in several layers of the starchy endosperm cells. However, *GUS* reporter gene expression was also detected in the maternal vascular tissue adjacent to ETC and vascular tissue of the lemma and palea (Li et al., 2008). Surprisingly, in barley the promoter of *OsPR602* was activated only in ETC and adjacent layers of starchy endosperm and the temporal and spatial patterns of *GUS* expression were perfectly correlated with the expression of the *END1* gene from barley and *TaPR60* from wheat (Doan et al., 1996; Kovalchuk et al., 2009). These data again suggest possible differences in ETC-specific**gene regulation between barley and rice.

The most probable roles of END1-like proteins in ETC are regulation of cell wall-ingrowth extension, formation of cellular membranes, lipid transfer to endosperm and/or defense from bacterial and fungal pathogens transported from maternal tissues.

## OTHER ETC-SPECIFIC GENES

Recently, a new ETC-specific gene, *AL1*, was isolated from rice (Kuwano et al., 2011; **Table 1**). The gene encodes a putative anthranilate N-hydroxycinnamoyl/benzoyltransferase and is expressed in the dorsal aleurone layer adjacent to the main vascular bundle. In rice, transfer cells are differentiated in this region. The role of this gene in plants remains unknown.

With the advent of new technologies for tissue/cell-specific transcriptome and proteome analysis (Thiel et al., 2012; Silva-Sanchez et al., 2013) it is expected that further genes with ETC-specific or predominant expression of novel function will be identified.

## POTENTIAL APPLICATIONS OF ETC-SPECIFIC GENES AND THEIR PROMOTERS FOR IMPROVEMENT OF GRAIN QUALITY AND YIELD

It is a well-documented that grain development in crops occurs under saturated supply of assimilates, which indicates that transportation of those nutrients from plant maternal tissues to embryo and endosperm is the main yield limiting factor (Borrás et al., 2004; Bihmidine et al., 2013). Therefore, manipulating the nutrients transport in order to increase grain sink strength is expected to lead to increased yield (Reynolds et al., 2009). Considering the role of ETC as a principal gateway regulating flux of nutrient precursors for endosperm filling, there is a huge, yet un-realized potential for engineering this gateway to increase grain yield and improve endosperm composition: quantity and quality of carbohydrates, proteins, lipids and micronutrients.

The importance of TCS for ETC development and the likely consequential involvement of TCS components in grain development, make this group of genes interesting tools for engineering or modifying grain quality and yield. Furthermore, involvement of TCS components in response to major abiotic stresses such as drought (Le et al., 2011; Kang et al., 2012), high salinity (Karan et al., 2009), and cold stress (Jeon et al., 2010) have been demonstrated. Although many genes representing the major components of TCS were recently identified in the barley ETC layer (Thiel et al., 2012), their function and responsiveness to environmental stresses and stimuli are largely unknown. Most of the existing studies on TCS-based cytokinin and ethylene signaling have been done in the model plant *A. thaliana*. Although studies have recently expanded to a broader range of plant species, there is still very little known about TCS function in cereals and specifically in grain development (Hellmann et al., 2010). Therefore, it is still too early to design or even predict possible applications of specific grain-related TCS genes in breeding and molecular genetics projects.

There are similar problems for biotechnologists interested in manipulating the transcriptional regulation of ETC development and function. Only one TF, ZmMRP-1, has been demonstrated to regulate ETC function (Gómez et al., 2002). Observations that ZmMRP-1 regulate several ETC-specific genes makes this TF a promising target. However, characterisation of transgenic plants with up- or down-regulated *ZmMRP-1* has not been reported. It may be that ectopic, constitutive overexpression of *ZmMRP-1* or silencing of this gene leads to significant pleiotropic changes in plant development or is lethal. It would be particularly interesting to express this gene under an ETC-specific promoter that is regulated by ZmMRP-1. This could lead to higher levels of ZmMRP-1 as a result of feed-back loop activation of the promoter. Higher levels of ZmMRP-1 could potentially enhance and/or extend in time the activation of target ETC-genes, and consequently further increase the development of cell wall-ingrowths (to increase cell membrane surface) and levels of ETC-specific proteins responsible for transport of lipids and sugars, hopefully culminating in increased efficacy of ETC function. An alternative to overexpression of *ZmMRP-1* could be manipulation of levels of ZmMRP-1 regulators, the MRPI proteins.

The best studied and currently most promising ETC-related genes for the engineering of grain quality and yield, and particularly for grain yield under stress conditions, are genes of sucrose synthases, INVs, and hexose transporters. There are considerable data on the structure and function of these genes (Weschke et al., 2000; Xu et al., 2012). Some of these genes are either specifically or predominantly expressed in ETC and tissues adjacent to ETC layers in barley (Weschke et al., 2000), sorghum (Jain et al., 2008b), cotton (Wang and Ruan, 2012), maize (Chourey et al., 2012; Liu et al., 2012), and rice (Wang et al., 2008a). Improvement of grain size and yield by overexpression of a hexose transporter has not been reported. Recent reports of the effects of sucrose synthase and cell wall-bound invertase overexpression, however, are astonishing (Wang et al., 2008a; Xu et al., 2012; Li et al., 2013). For example, overexpression of a potato sucrose synthase gene in transgenic cotton reduced seed abortion and increased the seed fresh weight by about 30% compared to the seed weight of control plants (Xu et al., 2012). Constitutive overexpression of the *Mn1 *gene from *Arabidopsis*, rice and maize in transgenic maize significantly increased invertase activities in leaves and developing seeds and dramatically improved grain yield through enlarged ears, and increased grain size and number (Li et al., 2013). Total starch content in transgenic kernels was also increased.

It is interesting however, that ectopic expression of cell wall-bound INVs does not always produce an expected positive effect on grain size and yield. For example, ectopic expression of the *GIF1* gene under 35S or rice *Waxy* promoters resulted in smaller grains, while overexpression of *GIF1* driven by its native promoter increased grain production (Wang et al., 2008a). The incorrect spatial or temporal expression of sugar INVs and hexose transporters can potentially lead to re-arrangement of auxin levels in grain tissues and consequential changes in auxin gradient. Decreased auxin levels impair development of ETC and other parts of endosperm (Bernardi et al., 2012). Therefore, knowing and considering the cascade of events induced by hormones during early stages of grain development is important for making correct decisions on spatial and temporal expression of genes, which can directly or indirectly influence auxin concentrations.

Another way to increase invertase activity, while maintaining the original spatial patterns of invertase gene expression, is in silencing invertase inhibitors (Rausch and Greiner, 2004). Silencing of invertase inhibitors in transgenic tomato plants resulted in an increased seed weight and increased levels of hexoses in fruit (Jin et al., 2009). Inhibitor(s) of ETC-specific INVs have not been reported, but if such inhibitor(s) exist, tissue-specific silencing of these gene(s) would be interesting to test.

It would also be interesting to express genes of sucrose synthase, invertase, and hexose transporters simultaneously, using stacking constructs and clever selection of suitable promoters. A simpler approach could be the tissue specific overexpression of a TF which co-ordinately regulates a group of sugar production and transport genes. It was recently reported that overexpression of the soybean *GmbZIP123* gene in transgenic *Arabidopsis* not only enhanced lipid accumulation in *Arabidopsis* seeds, but also up-regulated expression of two sucrose transporter genes and three cell-wall invertase genes by directly binding to their promoters. This in turn significantly increased levels of sucrose and both hexoses in seeds of transgenic plants compared to seeds of control plants (Song et al., 2013).

## CONCLUSIONS AND FUTURE PERSPECTIVES

ETC are highly specialized cells responsible for the delivery of signals and nutrients from maternal tissues to the developing endosperm. Large numbers of ETC-specific genes and genes predominantly expressed in ETC have been isolated and characterized from important cereal crop species and other plants during the last decade. Surprisingly, the most of identified genes fall into just four of the five groups. These four groups of genes are involved in signal transduction and/or transcriptional regulation in ETC. Genes from two groups are directly involved in transport of sugars and lipids to the endosperm. Because of the significance of ETC for grain development, ETC-specific genes and their promoters are important targets for the generation of transgenic crop plants with improved seed size and quality characteristics. The overexpression of ETC-specific *INCW* genes in transgenic rice and maize provides the first example of targeting ETC-specific genes for the manipulation of grain characteristics. However, a better understanding of the roles of ETC-specific genes encoding regulatory proteins is required for the correct application of these genes for grain biotechnology.

## Conflict of Interest Statement

The authors declare that the research was conducted in the absence of any commercial or financial relationships that could be construed as a potential conflict of interest.

## Footnotes

1 http://140.114.98.10/ltp/
